# Medical consumerism and the modern patient: successful ageing, self-management and the ‘fantastic prosumer’

**DOI:** 10.1177/0141076820911574

**Published:** 2020-09-10

**Authors:** Steve Iliffe, Jill Manthorpe

**Affiliations:** 1 Research Department of Primary Care & Population Health, University College London, London NW3 2PF, UK; 2 NIHR Policy Research Unit on Health & Social Care Workforce, King's College London, London WC2B 6LE, UK

## Introduction

Most doctors do not like medical consumerism. From its early days consumerism was ‘*an unwelcome thorn in the medical flesh*’.^1^ According to Downie,^2^ NHS patients simply cannot become consumers and doctors cannot become suppliers of goods and services. NHS patients consume services out of necessity (not want) and the state (not themselves) funds their care, so they are not customers.^3^ Medical services are, for the patient, an imperfect means to a desired end (like ‘good health’ or relief from pain), which is not a commodity.^4^ Patient-centred care has medical approval, but patients as consumers do not – as Gusmano et al.^5^ declare: ‘*Patient-Centred care, Yes; Patients as Consumers, No*’.

We understand and partially agree with these concerns and reservations, but we also see potential advantages in medical consumerism when it is defined as patient challenge to physician authority.^6^ In our view, medical consumerism has evolved through its encounters with medical services, producing different generations of consumers and changing definitions of consumerism. There is no such thing as medical consumerism in itself, but there are different forms of it which can combine in different ways.

We used a selective review approach to identify papers from medical, policy and health services research domains that contributed ideas and insights to the process of hypothesis generation. Synthesising this literature, we conceptualise three generations of medical consumerism. In the first generation, beginning in the USA in the 1960s, but spreading later to the UK, patients challenged professional authority.^6^ The response of the NHS to this challenge was to offer a menu of choices, for example, giving birth at home or in a midwife-run unit with or without a water bath. In countries with commercialised medical care, like the USA, these responses were driven by the need to compete for customers. In the NHS, patients do not pay directly or through insurance but increased custom could still be used by the service to negotiate additional resources from the NHS.

In the second generation, arising in the 1980s, self-funding consumers purchased their desires, mostly forms of body enhancement, in a burgeoning niche market. Second-generation medical consumers are commodified – their bodies have exchange value, and the customer (not an insurance policy or a public health service) pays.

The third generation of medical consumers was co-opted into healthcare systems starting in the 1990s, as market mechanisms became the favoured model for healthcare organisations, to help contain costs and increase productivity; its consumers are ‘disciplined’.^7^ This third generation has evolved special forms of disciplined consumerist behaviour, three of which we describe in this paper: successful ageing; self-management; and prosumerism (blending production and consumption).

**Figure 1.**
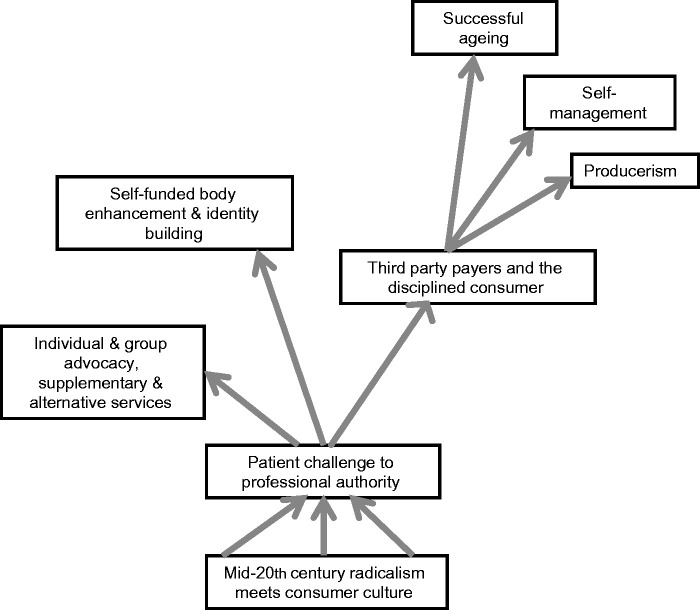
The exuberant growth of medical consumerism.

The relationships between these generations are summarised in Figure 1. Although they emerged at different times, all three generations are still having effects on health services and patients, to differing extents at different times, in different places. As far as we can see, the consumerist generations do not succeed each other but co-exist.

## First generation: patient challenge to professional authority

The expansion of medical consumerism coincided with the introduction of science-based medical technologies (like X-rays) and therapies (like Penicillin and its successors) immediately after World War II. Medical consumerism emerged first in the USA, which was leading the growth of medical science, then spread to the UK and beyond.

Consumerism's original role was to protect citizens against sellers’ risks and failings, which were widespread in the American healthcare system.^8^ The medical consumer had genuine grievances. Medical care created a ‘*human rights wasteland*’ where many patients were not consulted about important treatment decisions, hospital incarceration lacked legal protections, while some were experimented on without their consent.^9^

This paternalism provoked resistance, particularly from the emerging Disability Rights Movement, and this resistance fostered the introduction of informed consent prior to treatment, legal frameworks for compulsory assessment or treatment, and better safeguards for research involvement. The Women's Movement also influenced medical consumerism around reducing professional control of women's bodies, and challenged professional assumptions and beliefs about abortion, childbirth and breast cancer treatments.^10^ Sympathetic professionals sided with consumerism; as one US doctor put it ‘*us professionals need watchdog organisations to keep us clean, focussed and less arrogant*’.^7^

In the beginning, medical consumerism took up the tasks of creating information networks and special interest groups, offering patients mutual support^8^ and in some places launching services, like free clinics, that competed with the existing medical system.^11^ Its appeal was greatest when services were simple, and choices were mostly about preferences. Examples are childbirth, which continues to change under consumer pressure, and the right to assisted dying, which is currently challenging professional and public opinion.

Although first-generation consumerism undermined insular professional decision-making and opened up US hospital management boards to wider publics,^10^ its limitations soon became apparent. People who were consumerist in attitude when well often became more passive and less critical when ill, while first-generation consumerism dented medical authority less than its proponents expected. Nevertheless, medical consumerism encountered professional hostility. One review concluded that patient challenges to physician authority worsened doctor–patient relationships, lead to prolonged and conflictual encounters, and reduced treatment concordance.^12^ The only positive impact noted was a possible improvement in professional decision-making.

Medical consumerism, according to Zeckhauser and Sommers,^12^ is a classic Principal–Agent problem. The consumerist patient (principal) wants a unique, personalised assessment of their problem and wants to express preferences. The professional (agent) wants consultations to stay within time limits and uses professional reasoning rather than the patient's information to make decisions and recommendations. Neither may be satisfied by their encounter.

In Habermas’ terms, the first generation of medical consumerists struggled with consultations that were ‘concealed strategic actions’ containing either unconscious deceptions (systematically distorted communication) or conscious, manipulative communications. Or, putting it another way, encounters were unsatisfying because communication was not comprehensible, not true, not appropriate (to the situation) or insincere.^13^

First-generation medical consumerism, while widespread, is too weak to evaluate and change healthcare, to the regret of those promoting health service marketisation. In the USA, the hope that, as patients ‘*become more sophisticated purchasers of health care, they will push competition in health care delivery to look increasingly like that in consumer-goods industries*’,^14^ has not materialised. The tools that consumers need to judge the quality of services, like clinical safety, user satisfaction and cost, are not adequate for the task. Few consumers view hospital IT portals and even fewer enter them. When US patients have insurance cover without substantial co-payments, then consumerist activity declines because there is no incentive to ‘shop around’ on cost.

Since the 1980s, UK health consumer movements have felt it necessary to defend not criticise the NHS, with a visible evolution from challenging medical dominance to participating in ‘managed consumerism’.^15^ We will return to ‘managed consumerism’ as the defining characteristic of third-generation consumerism.

Consumerism is evidence of late modernity's reflexive self; patients acting in a calculating way engage in self-improvement while being sceptical about expert knowledge. The underlying assumption of the first generation of medical consumerism is that good quality medical care arises from challenges buttressed by the possibility of external sanctions, not from intrinsic trust or cooperation.

However, we are increasingly recognising the changeable nature of the complex desires, emotions and needs that characterise patient–professional relationships. People may express ideal-type ‘consumerist’ behaviour and the ‘passive patient’ position simultaneously or variously in their interactions with professionals, depending on context.^16^ First-generation consumers wanted more information but did not want to use it to make decisions, which they regarded the professional's job.^17^

None of this is surprising. Research points to the remarkable persistence of asymmetry in the knowledge held by the professional and the patient. Such asymmetry is at the core of medicine, and managing it is what doctors do. Those who have mounted patient challenges to professional authority, in the expectation that knowledge asymmetry can be overcome, are misunderstanding the role and nature of medicine.^18^ To put it another way, the transformative power of the first-generation medical consumer has been over-estimated.

Nonetheless, patient challenges to professional authority persist, as the case of Charlie Gard shows (see Box 1).

**Box 1.** The case of Charlie Gard.^19^

Charlie Gard was a baby who had a rare disease – infantile onset encephalomyopathic mitochondrial DNA depletion syndrome – which damaged his brain and resulted in him needing mechanical ventilation at Great Ormond Street Hospital, in 2017. Paediatricians recommended palliative care and withdrawal of the ventilator.
Charlie's parents opposed this plan and raised $1.6 million from public donations to take him to the USA, where a specialist had developed an as yet untried treatment for a similar but not identical condition. A campaign group, Charlie's Army – slogan ‘If he's still fighting we are still fighting’ – supported the parents with a 350,000 signature petition.
Paediatricians and other hospital staff received hostile messages and some death threats, the Pope and Donald Trump intervened to offer Charlie support, and four courts (three in the UK plus the European Court of Human Rights) supported the hospital's argument that further treatment would not be in Charlie's interests. There were angry scenes outside the Courts and hospital. Charlie died just before his first birthday.
According to Sky News, the parents said their son could have been a ‘normal, healthy boy’ if their attempts to access experimental therapy had not been blocked by the courts.

For all its weaknesses, the legacy of first-generation medical consumers that emerged in the 1960s survives; supporting individuals to cope with their situations, offering alternative therapies and becoming increasingly accessible through IT and social media. One example of this persistence that provokes medical hostility is the growing resistance to vaccination (especially, but not exclusively, against measles, mumps and rubella). Other examples that are perceived more benignly are support and campaigning networks like Maternity Action and the National Childbirth Trust that have evolved around pregnancy and childbirth.

## Second generation: self-funded body work and identity building

When the patient has money, then medical consumerism can expand into the area of body enhancement, which draws on the ‘pedagogy of defect’ which teaches (mainly) women that their bodies are faulty and unacceptable. This is second-generation consumerism. Enhancements can be surgical (cosmetic surgery), pharmacological (mood-altering drugs like ‘Prozac’), genetic (genetic engineering) and (coming soon) cybernetic.

Second-generation medical consumerism thrives in the USA, where 18 million people underwent cosmetic surgery procedures in 2018.^20^ North American plastic surgeons are increasingly competing with each other and driving down cosmetic procedures’ prices.^21^ This fits with Baudrillard's judgement^22^ that ‘*the system of needs is the product of the system of production*’, which we can also call ‘supplier-induced demand’.

The UK may not follow the US trend. After a decade of near consistent growth, there were over 28,000 cosmetic procedures in the UK in 2017, 40% down from the peak in 2015.^23^ This fall was attributed to shifts in mood in social media, which had responded to criticisms about the need to portray more diversity among people of different sizes and shapes. In the UK, there seem to be limits to supplier-induced demand. For example, cosmetic surgeons in the UK have debated a ban on advertising – as occurs in France – as a way of minimising harms from unskilled or inappropriate procedures.

Professionals consulting second-generation consumers, no longer hapless victims of fate but active agents grappling with their circumstances, fall easily into Baudrillard's ‘*alibi of individual needs*’,^22^ whereby the person's desire for enhancement is presumed to be a consequence of prior need. Baudrillard argued instead that production creates need by feeding the culture of bodily defectiveness with promises of improvement. Everyday life is changed by enticing technical solutions to bodily problems. In Habermas’ terms, the ‘*life-world*’ (everyday life) is colonised by science.^12^

The professional encountering a consumer demand that seems inappropriate has three choices: to accept the consumer's demands and proceed; to invoke medical expertise and argue that the desired enhancement is not applicable or even dangerous; or to claim that their conscience forbids collusion.^24^ Between these positions, dialogue is possible but arduous.

The second-generation consumer can opt out of negotiations with reluctant doctors or health services by turning to medical tourism. Medical travel agencies explain that cosmetic surgery is accessible to all, not just the global elite, aligns its recipients with people of worth and offers multiple choices.

## Third generation: the disciplined consumer

When health service costs are constrained, medical consumerism changes again. The fundamental characteristics of the third-generation consumer are personal responsibility, proactive and prevention-conscious lifestyle behaviours, rationality in decision-making, and exercise of choice. Health services encourage consumers to learn more about their choices and to exercise them cost-consciously.

In England, choice is built into accessing services (choosing a health centre or dentist to register with, booking a GP appointment, selecting a hospital for specialist care) while judging the quality of services using publicly available knowledge from the Care Quality Commission. The shift from patient to medical consumer puts the responsibility for medical decisions, choosing wisely, and their outcomes on those seeking help, guidance and care,^25^ both in the ‘managed consumerism’ of the USA^26^ and England's ‘partnership’ approach.^27^

While first-generation medical consumerism emphasised system problems, third-generation consumerism is individualised within a person-blame framework.^25^ This shift in consumer role serves the interests of a medical system that seeks to expand its patient base while reorganising its services to contain costs. Choice is coupled to processing patients, redesigning systems and meeting targets. Third-generation consumers are pro-active in seeking help from a health system that idealises the benefits of screening, offers urgent and aggressive therapies and promotes faith in medical science.

In 2002, Wanless^28^ argued that the viability of the NHS depended on its ‘*full engagement*’ with the public. The ‘*fully engaged*’ individual becomes responsible for maintaining their own health, preventing or at least postponing illness and disability by lifestyle modifications (successful ageing – see below), self-managing any long-term conditions, and at the extreme, producing individualised services and then consuming them as a ‘prosumer’. We consider each of these methods of engagement in turn.

### Successful ageing

Successful agers are satisfied, active, independent, self-sufficient and, above all, challenge decline, making them optimistic about self-directed health. Rowe and Kahn,^29^ the parents of ‘successful ageing’, describe it in consumerist terms:

*To succeed … means having desired it, planned it, worked for it. All these factors are critical to our view of aging which … we regard as largely under the control of the individual. In short, successful aging is dependent upon individual choices and behaviours. It can be attained through individual choice and effort*.

This view fails to acknowledge the impact on health of power relations in an ageing society, the social determinants of health and widening inequalities.

### Self-management

Emphasising patient responsibility, and acting in concert with providers, self-management seems a promising strategy for managing long-term conditions, – moving beyond education to teaching individuals to actively identify and solve problems associated with their illness. Self-management also shows potential in prevention by encouraging healthy behaviours and strategies for managing symptoms.

Self-management should attract the third-generation consumer and has been part of NHS policy since the Expert Patient Programme was imported from California in 2002, but evidence of benefit is thin. For example, the National Diabetes audit for 2015/16 reported that 77% of eligible patients were referred to a structured education programme but only 7% of them actually attended.^30^

### Prosumerism

The melding of production and consumerism into ‘prosumerism’ promises – for some – liberation and empowerment. The prosumer decides what s/he needs, produces it and then consumes it. When Toffler introduced the idea of prosumerism in 1980,^31^ it was presented as a fantastic revolutionary activity that would transform the economy and everyday life. Its impact on medical consumerism has been modest.

In England's health and social care system, prosumerism is currently exemplified by Personal Budgets and Personal Health Budgets. In a Department of Health briefing published in 2008,^32^ an older person creates and then consumes a customised care package (see Box 2).

Personal Health Budgets are controversial. Williams and Dickinson^33^ see Personal Health Budgets as encouraging a transactional relationship based on individual entitlements that may exacerbate persistent gaps between demand and resources. As Mold notes,^34^ individual choice tends not to be so prominent a demand as greater autonomy, the ability to complain, receiving more information, and protection of patients' rights.

**Box 2.** A case study in prosumerism (adapted from Department of Health 2008).

June suffers from lung disease, a condition which varies from day to day which impacts greatly on her level of independence at home. She was very isolated, couldn't cope with domestic tasks or any paperwork, couldn't get out without support and was depressed.
The only thing she wanted was the company of a mature lady to act as a Personal Assistant as and when she needed, and who would be willing to drive her around. The (social care) agency was commissioned to provide her with a number of hours of support per week that she could use to meet the outcomes of her support plan.
A year later, she gets personal care only when her health condition is deteriorating and she feels pretty much in control of her life, is not depressed anymore and has used her support to visit garden centres, combine shopping with a visit to a café and go to a Christmas pantomime. She prepared all necessary documents, went shopping and packed her luggage for a short cruise (gift of her daughter) and has coordinated the spring cleaning before the Housing Association (landlord) revamps her kitchen.
She gets more visits from her daughters because now she has positive things to say and does not complain as much.

Despite these critical views about prosumerism, it resonates with current times. The economist Robin Murray, discussing Zuboff and Maxim's book ‘*The Support Economy?*’ highlighted their vision of the individual at the centrepiece of the economy, who like ‘*any producer needs all sorts of systems, tools, knowledge, advice etc. suited to their specific needs*’. The implications for health are that:

*… you can't just have an NHS advisor with a script, who is low down on the hierarchy, you have to have someone who is, as it were, your partner – who then scours the world and helps you make your decisions*.^35^

## Discussion and conclusions

The culture wars around medical consumerism will be complicated and lengthy. Medical consumerism is ubiquitous in the UK, where its first generation promoted a rich matrix of self-help organisations, its second generation stimulated development of niche markets and its third engaged citizens with the NHS.

Asymmetry between patients and professionals will not decrease because modern medicine's ‘*intensity and incomprehensibility*’ will generate new questions and demands for clarification.^25^ The challenge for both patients and professionals is to shape this new dialogue, and to reduce Habermas’ ‘*systematically distorted communication*’. The need for a number of research, development and policy initiatives is clear. First, we need to know if the systematically distorted communications noted in first-generation consumerism have weakened in the last 20 years. This may be difficult given that it requires retrospective evaluation methods that are open to bias, but it is also an opportunity to revisit archives of qualitative data for historical evidence. We also need to acquire evidence about the comprehensibility, truthfulness, appropriateness and sincerity of today's consultations, some of which will be by phone or email, which may not be analysable using methods appropriate to recorded speech and visual interpretations. There is some urgency here to develop methods of understanding doctor–patient communication, particularly when health services are encouraged to push back against some consumers (for example, the ‘anti-vaxxers’) as some people advocate. To complicate the situation, outcomes in different settings (for example, the rural outpatient clinic compared with the teaching hospital), different systems (the NHS in the devolved nations) and different disciplines (for example, general practice where relational expertise predominates compared with neurosurgery where technical expertise predominates) may differ.

Frank^24^ encourages us to contest the definition of ‘needs’ and to refuse others’ utopias, instead taking stock of who we are. We will experience this when our child, for example, expresses a desire to have braces to straighten their teeth or wants a tattoo (acquired on holiday) removed. We will need to develop a dialogue which allows families and friends to talk about the kind of florid second-generation consumerism that now appears on television and especially online. NHS professionals will benefit from being able to talk about body enhancement and identity with their patients. Where production creates needs, we must think about limiting production, perhaps by introducing a French-type ban on advertising cosmetic surgery, without forgetting that work on communication skill-sets is likely to be more important than legislation.

Third-generation medical consumers have made a pact with the medical system, re-energising medical science's colonisation of the lifeworld^25^ in return for some choice in place of treatment or treatment preferences. Wanless’ gloomy prognostication^28^ looms over us; if the population does not fully engage with the NHS, the service will fail under the strain of demands from passive consumers. There are risks here that doctors will blame patients for failing to age successfully, to manage their long-term conditions or to take up the prosumer role. If your patient has a stroke or a coronary, some may think it was their own fault for not aligning personal behaviour with their ‘true’ (that is, disciplined) selves. It would be surprising if at least some politicians decided to blame doctors for not managing consumerism forcefully enough. Professional development will need to include ways of mitigating these risks.

The evolving forms of medical consumerism suggest to us that a thoughtful, decisive and frugal third-generation consumer is emerging with positive attributes which should be nurtured. There are implications for professionals but also politicians in this. Ageing successfully depends on socioeconomic circumstances and personal history as well as on individual lifestyle. Self-management is realisable, up to a point, but will need substantial resources if all those with long-term conditions are to become lay experts with enough expertise to improve outcomes. And ‘fantastic prosumers’ may turn out to be atypical examples of medical consumerism, rather than its vanguard.

## Declarations

### Guarantor

SI.
